# Understanding the Nanoindentation Edge Effect of Single-Crystal Silicon Using Molecular Dynamics Simulations

**DOI:** 10.3390/mi16070814

**Published:** 2025-07-16

**Authors:** Chao Long, Ruihan Li, Pengyue Zhao, Ziteng Li, Shuhao Kang, Duo Li, Huan Liu

**Affiliations:** 1 Center of Ultra-Precision Optoelectronic Instrumentation Engineering, Harbin Institute of Technology, Harbin 150001, China; 24s008073@stu.hit.edu.cn (C.L.); zhaopengyue129@126.com (P.Z.); 2 College of Mechanical and Electrical Engineering, Harbin Engineering University, Harbin 150001, China; ruihanli@hrbeu.edu.cn; 3 State Key Laboratory of Mechanical System and Vibration, School of Mechanical Engineering, Shanghai Jiao Tong University, Shanghai 200240, China; 22s108282@stu.hit.edu.cn (Z.L.); xiaoshe0614@sjtu.edu.cn (S.K.); duo.li@sjtu.edu.cn (D.L.)

**Keywords:** molecular dynamics, single-crystal Si, nanoindentation, edge effect

## Abstract

The edge effect refers to what occurs when an object undergoes elastic contact with the edge of a material. This is common in practical applications, but the understanding of this phenomenon is not yet mature enough, and understanding the microscopic characteristics of the material regarding this phenomenon is necessary. This article investigates the edge effects of single-crystal silicon at different indentation positions through molecular dynamics simulations. The results indicate that the edge effect of the indentation is influenced by the indentation position and depth. The closer the indentation head is to the edge of the workpiece, the more particles are extruded from the side of the workpiece and the wider the collapse range of the indentation surface. At the same time, the indentation position also affects the distribution of the von Mises stress and phase transition area. When the edge effect occurs, the von Mises stress and phase transition region tend to be concentrated near the workpiece edge. This study demonstrates the atomic-scale deformation mechanism of single-crystal silicon under varying indentation positions.

## 1. Introduction

Since its discovery, single-crystal silicon has been extensively utilized in both the semiconductor industry and microelectromechanical systems [1,2,3,4]. Its exceptional attributes, including high wear resistance, thermal stability, and resistance to corrosion [5], have enabled it to dominate the electronic consumer market [6]. Concurrently, there is an increasing demand for smaller electronic devices, necessitating the integration of more components within limited space [7]. Consequently, investigating the nanoscale deformation mechanism of silicon is essential for gaining deeper insights into its characteristics [8,9].

Nanoindentation serves as a key technique for characterizing the properties of materials at the micro-/nanoscale [10,11,12]; the tool tip pressing into the material surface causes the material to experience normal force and shear force during the nanoindentation experiment, which is similar to the actual processing conditions of the materials [13,14]. Therefore, nanoindentation has become a widely adopted method for analyzing the microscale deformation of materials. Scanning tunnelling microscopy (STM) and transmission electron microscopy (TEM) are frequently employed to analyze structural changes in materials following nanoindentation tests [15,16,17]. Nevertheless, challenges persist in the real-time monitoring of deformation mechanisms and internal defect evolution during the indentation process, imposing limitations on advancements in material science [18]. The deformation and phase transition mechanism of materials during indentation can be observed through atomic-level simulation [19]. Zhao et al. [20] used ABAQUS to establish finite element models for monocrystalline silicon at room temperature and high temperature; they concluded that the hardness of single-crystal silicon workpieces was lower at high temperature compared to room temperature, and at the same time, they carried out nanoindentation tests to validate the accuracy of the findings. Heidari et al. [21] studied the effect of particle distribution on materials through nanoindentation experiments and FEM simulations and found that high-heterogeneity materials require direct contact area measurement to improve testing accuracy.

Molecular dynamics (MD) is a method of simulating material changes during processing at the atomic level, and it has been applied in nanoscratching and nanocutting [22,23,24,25]; additionally, MD simulations have been utilized to analyze the mechanical behavior during nanoindentation across various metals, including gold [26,27], aluminum [28,29], nickel [30,31], and so on. Lin et al. [32] investigated the phase transition behavior of silicon during the nanoindentation of single-crystal silicon using MD simulation. They found that when the indentation load was small, the material underwent elastic deformation and recovered after unloading. When the load was too large, the material underwent plastic deformation and did not recover after unloading due to the generation of a permanent amorphous phase. Chung et al. [33] conducted an MD simulation study on the deformation behavior of single-crystal silicon during nanoindentation. Their research, which focused on the influence of indenter semi-angles, employed three different angles for investigation. The results demonstrated that with an increase in the semi-angle, both the material hardness and elastic modulus exhibited a gradual decline. In another study, Jiao et al. [34] performed MD simulations to examine the nanoindentation process of single-crystal silicon at an extremely low temperature of 1 K. Their work aimed to investigate the phase transformation mechanisms of monocrystalline silicon under such low-temperature conditions. The findings revealed that the indentation depth plays a significant role in determining the internal structural evolution of the material. Ge et al. [35] applied machine learning to MD simulations to study phase transitions in silicon nanoindentation. They employed machine learning techniques to develop a set of accurate atomic potential functions for simulation. Their study revealed that the geometrical configuration of the indenter could substantially influence the material’s structural characteristics following unloading.

The edge regions of electronic devices exhibit lower hardness compared to their central areas, making them more susceptible to damage. Consequently, it is essential to investigate the mechanical properties of material edges. When the indenter establishes elastic contact with the edge of the material, an edge effect is observed [36]. Jakes et al. [37] utilized the standard Oliver–Pharr method to evaluate nanoindentation data for fused silica, poly(methyl methacrylate), and poly(methyl methacrylate) samples, investigating the edge effect’s influence on these materials. Their findings revealed that material properties significantly affected the edge effect, with hardness decreasing as the indentation position approached the edge. Liu et al. [38] carried out MD simulations focusing on the edge effects in gallium nitride during nanoindentation. Their research indicated that as the distance between the indenter and the edge of the workpiece decreased, there was a gradual increase in the number of particles being ejected from the sample’s side. Furthermore, they observed that both the von Mises stress and the indentation force were significantly influenced by the specific position of the indentation. Li et al. [39] performed MD simulations focusing on the edge effect in single-crystal silicon. Their primary investigation centered on the temperature-dependent edge effect under constant-indentation conditions. Through experiments at six distinct temperatures, they observed that elevated temperatures led to an increase in the quantity of side-stacking material, accompanied by a reduction in indentation load. However, the position and depth of the indenter during edge effects in single-crystal silicon remain uncertain, which is crucial for understanding the lifespan of components fabricated from single-crystal silicon.

The primary objective of this study is to examine how the indentation position influences the edge effect in single-crystal silicon during the nanoindentation process. Using MD simulations, a series of nanoindentation process models were developed to evaluate the impact of the indentation head position and depth on the material’s lateral surface area and surface collapse behavior. This article systematically analyzes the morphology of indentation positions on the surface of workpieces at different positions, changes in internal defects and dislocations of workpieces, as well as changes in forces and subsurface stresses during cutting processes. This work helps to comprehensively understand the edge effects of single-crystal silicon from a microscopic perspective.

## 2. Method

**Figure 1:**
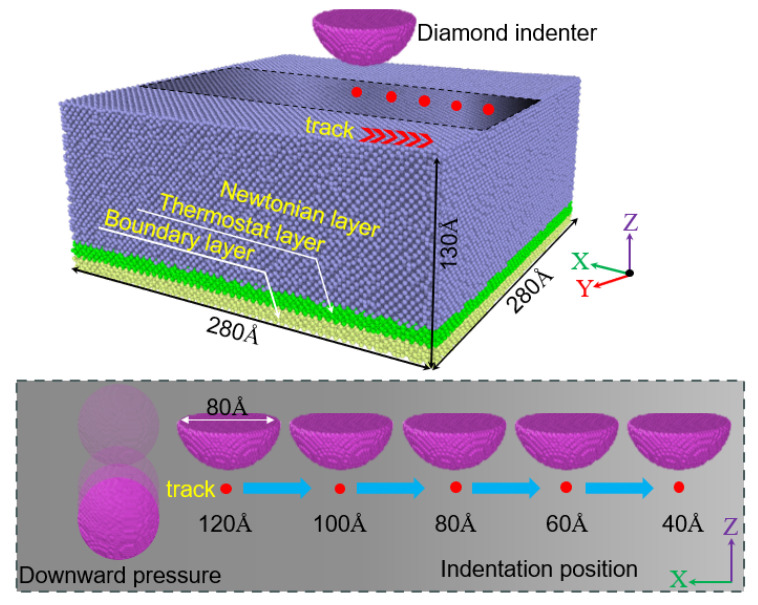
MD model of single-crystal silicon nanoindentation.

This study employs the MD method, utilizing the LAMMPS open-source platform, to investigate the edge effects of nanoindentation on the surface of single-crystal silicon. Figure 1 shows the MD simulation nanoindentation model we established, which comprises a single-crystal silicon workpiece and a diamond indenter. The dimensions of the single-crystal Si workpiece are 28 × 28 × 13 nm^3^, with a total of 514,200 atoms. The crystal orientation of single-crystal silicon has an excellent comprehensive performance of <1 0 0>. The diamond indenter, with a radius of 4 nm, is composed of 47,104 atoms. In the single-crystal silicon workpiece, the atoms are categorized into three distinct regions: the Newtonian layer, the thermostat layer, and the boundary layer. The Newtonian layer, where atoms move in accordance with Newton’s laws of motion synchronized with the indenter, is the primary region for dislocation formation and phase transformation within the workpiece [40]. The indentation process generates heat that raises the temperature of the workpiece. The stability of the system temperature can be maintained by releasing heat through the atoms in the thermostat layer [41]. In this simulation, we did not investigate the effect of temperature on the edge effect of indentation, so the temperature of the thermostat layer was set to 293 K. The atoms within the boundary layer are held stationary, ensuring the stability of the nanoindentation system [42]. We use free boundaries in the X and Z directions, which can better help us observe edge effects. The use of periodic boundaries in the Y direction is mainly to eliminate size effects and ensure the feasibility of calculations. We conducted five simulations of the nanoindentation model, with distances of 120, 100, 80, 60, and 40 Å from the material edge to the indentation head. In this study, we used the distance from the indentation head to the material edge to represent the nanoindentation position.

As shown in Table 1, the Tersoff potential function was selected for modeling the interaction forces between silicon atoms and between silicon and carbon atoms in the nanoindentation process of single-crystal silicon. This choice was made because the Tersoff potential explicitly accounts for the effects of covalent bonding [43]. Meanwhile, dynamic pre-relaxation was used in the NPT ensemble to pre-relax the system before nanoindentation, achieving equilibrium at 293 K [44]. The indenter performs nanoindentation on the single-crystal silicon workpiece at six distinct positions, with an indentation speed of 50 m/s [6]. The workpiece is pressed at 4.6 nm with an integration step size of 1 fs. Moreover, we studied the indentation force and von Mises stress during the indentation process, and the formula for the indentation force is shown in Equation (1) [45], and the formula for the von Mises stress is shown in Equation (2) [46].

(1) $$F=\frac{4}{3}E_{r}h^{\frac{3}{2}}\sqrt{R}$$

(2) $$\sigma_{vm}=\sqrt{\sigma_{xx}^{2}+\sigma_{yy}^{2}+\sigma_{zz}^{2}-\sigma_{xx}\sigma_{yy}-\sigma_{yy}\sigma_{zz}-\sigma_{zz}\sigma_{xx}+3(\sigma_{xy}^{2}+\sigma_{yz}^{2}+\sigma_{zx}^{2})}$$

The nanoindentation model code is run by LAMMPS and the simulation results are visualized using the Ovito software with the version number of 3.12.2 [47,48].

## 3. Results and Discussion

### 3.1. Surface Deformation Mechanism

**Figure 2:**
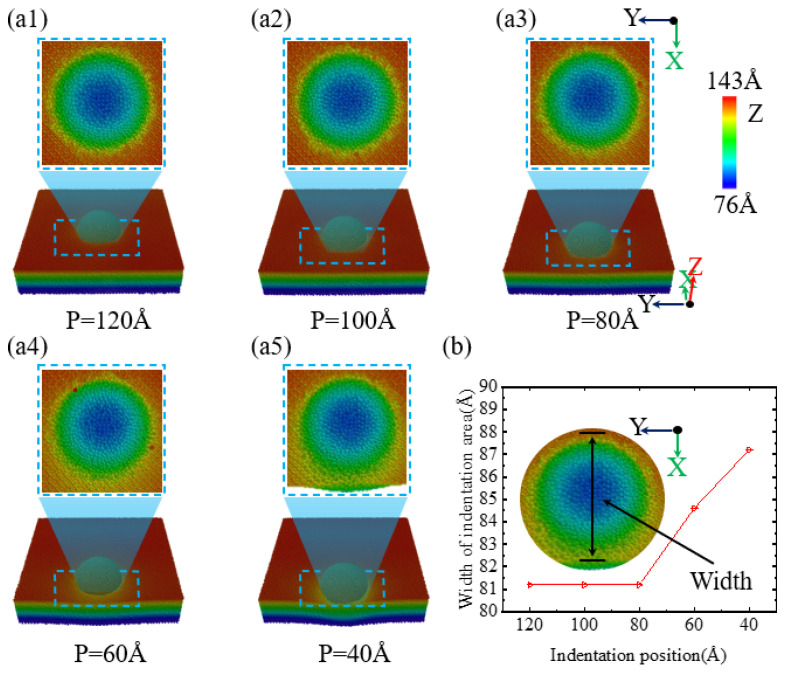
The surface morphology changes in a single-crystal silicon workpiece at an indentation depth of 4.6 nm at different indentation positions. (**a1**–**a5**) Surface morphology at different indentation positions; (**b**) width of the indentation area at different indentation positions.

During the nanoindentation of single-crystal silicon, the position of the indentation greatly influences the morphology of the indented region. Figure 2(a1–a5) illustrate how the morphology of the indented area changes with varying indentation positions. When the indentation positions are 120 Å, 100 Å, and 80 Å, as shown in Figure 2(a1–a3),b, the indented area exhibits a circular collapse with a diameter of 80 nm, and the depth of the collapse is generally consistent at 81.25 Å. However, when the indentation position is further moved in the -X direction, that is, towards the edge of the specimen, as illustrated in Figure 2(a4), at an indentation position of 60 Å, a slight collapse begins to appear along the -X direction at the edge of the indentation, though the collapse depth does not show significant change. As depicted in Figure 2(a5), a progressive increase in the collapse area along the -X axis is observed at 40 Å as the indentation point approaches the sample’s perimeter. Simultaneously, the collapse depth exhibits a substantial rise. Furthermore, Figure 2b illustrates that at an indentation depth of 4.6 nm and for positions at or beyond 80 Å, the morphology of the indented area on the specimen surface remains the same. However, when the indentation position is less than or equal to 60 Å, the collapse area of the indentation on the single-crystal silicon specimen surface increases as the indentation position moves closer to the edge of the specimen.

**Figure 3:**
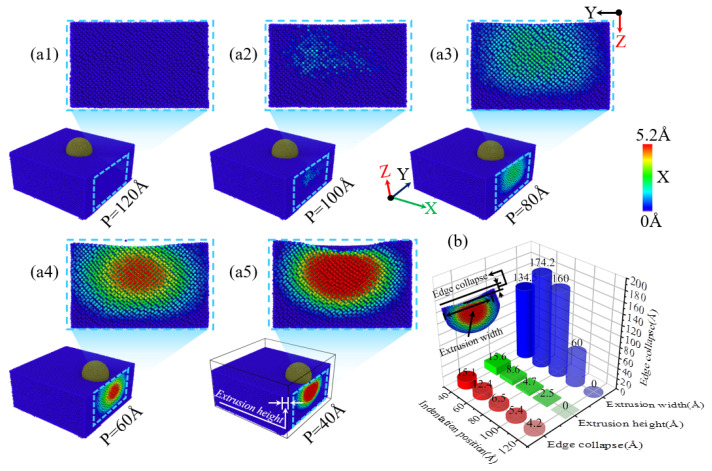
The changes in material extrusion and edge collapse on the side of the workpiece at different indentation positions with an indentation depth of 4.6 nm. (**a1**–**a5**) Side view morphology at different indentation positions; (**b**) bar charts of the material extrusion area width, height, and collapse depth at different indentation positions.

During the nanoindentation of single-crystal silicon using a diamond indenter, the location of the indentation plays a role in the material’s extrusion behavior along the specimen’s edge. Figure 3(a1–a5) illustrates the resulting extrusion morphologies observed on the single-crystal silicon specimen’s side at a consistent 4.6 nm indentation depth but with varying indentation positions. At a 120 Å indentation position, the specimen’s side maintains its smoothness, and no material extrusion is observed, as depicted in Figure 3(a1). However, at an indentation position of 100 Å, as shown in Figure 3(a2), a small amount of slight material extrusion begins to appear. As the indenter moves further towards the -X direction of the specimen edge, as illustrated in Figure 3(a3), at an indentation position of 80 Å, there is noticeable material extrusion with a larger extrusion area and a significantly increased extrusion height. As the indenter approaches the -X direction edge of the specimen further, as illustrated in Figure 3(a4),b, as the indentation position moves towards the edge, it can be observed that the width and height of the material extrusion at the edge of the specimen show a gradually increasing trend. However, compared to the indentation position of 80 Å, when the indentation position is 60 Å, as shown in Figure 3(a4,a5),b, the extrusion area on the specimen’s side decreases while the extrusion height increases significantly as the indenter moves further towards the -X direction of the specimen edge, and the collapse area at the indentation position on the specimen surface also shows an expanding trend. As illustrated in Figure 3b, with the indentation position moving closer to the -X direction edge of the specimen, the depth of collapse in the edge region increases progressively, with a more significant increase at an indentation position of 60 Å, and the extrusion height also gradually increases, with a more significant increase at 40 Å. Additionally, the change in the width of the material extrusion on the side of the specimen is also closely related to the indentation position. For indentation positions of 120 Å, 100 Å, 80 Å, and 60 Å, as the indentation position approaches the edge region, the width of material extrusion on the side of the specimen shows an increasing trend, but at 40 Å, the width of the material extrusion significantly decreases. In addition, the morphology of the extruded side of the material is elliptical, which is consistent with the simulation results of previous scholars and is caused by the diamond cubic crystal structure of single-crystal silicon [39].

**Figure 4:**
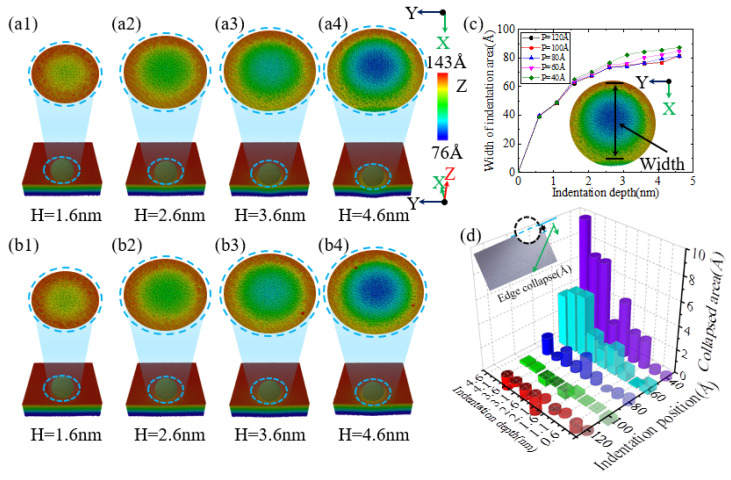
The changes in surface indentation and edge collapse of the workpiece at different indentation depths for indentation positions of 40 Å and 60 Å. (**a1**–**a4**) Indentation at a position of 40 Å; (**b1**–**b4**) indentation at a position of 60 Å; (**c**) width of the surface indentation area at different depths and indentation positions; (**d**) surface height difference at different depths and indentation positions.

When performing nanoindentation experiments on the edge of single-crystal silicon using a diamond indenter, the experimental results show that the indentation depth has a significant impact on the edge effect. As illustrated in Figure 4(a1,b1),c, when the indentation depth is 1.6 nm, the indentation area sizes at positions 40 Å and 60 Å are similar. However, when the indentation depth reaches or exceeds 2.6 nm, as illustrated in Figure 4(a2–a4,b1–b4), as the indentation depth increases, the area of the indented region on the specimen surface also expands accordingly. This is mainly because the indenter is spherical; when the indentation depth is less than the indenter radius, an increase in indentation depth will lead to an increase in the contact area between the specimen and the indenter, resulting in a larger indentation area, consistent with previous research on nickel edge effects [49]. In addition, at an indentation depth of 4.6 nm, an obvious collapse phenomenon appeared at the edge of the indentation area on the specimen surface. Figure 4c shows the curve of the indentation area width on the specimen surface as a function of indentation depth for different indentation positions. As illustrated in Figure 4c, when the indentation depth is no less than 2.6 nm, the indentation area on the specimen surface increases with increasing depth, and at positions 40 Å and 60 Å, the area increase is particularly significant. Figure 4d shows the variation in the height difference on the specimen surface under different indentation positions and depth conditions. It can be observed that when the indentation position is greater than or equal to 80 Å, the height difference value on the specimen surface is relatively small and exhibits irregularity. However, as the indentation position gradually approaches the edge of the specimen, especially at positions 40 Å and 60 Å, the height difference value on the specimen surface increases significantly, which strongly proves the existence of the edge effect in single-crystal silicon materials.

**Figure 5:**
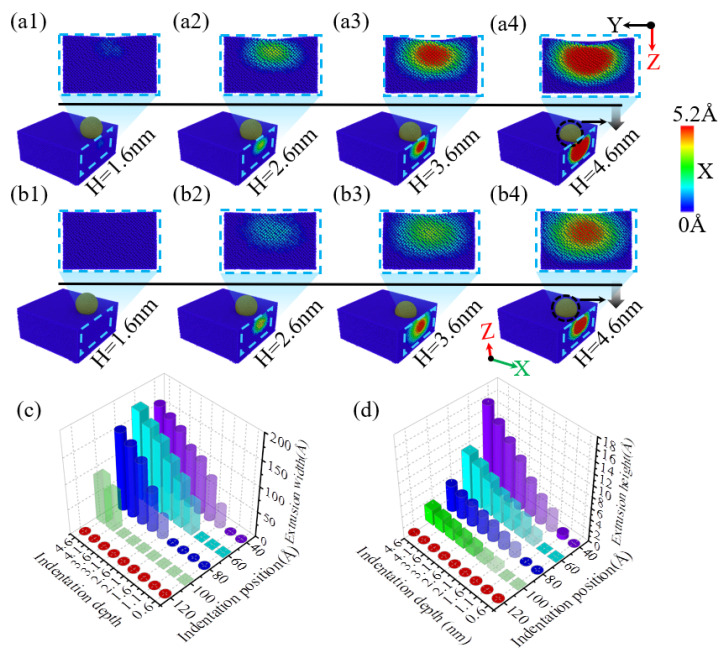
The material extrusion on the side of the workpiece during nanoindentation of single-crystal Si at different indentation depths. (**a1**–**a4**) materials extrusion with indentation position of 40 Å; (**b1**–**b4**) materials extrusion with indentation position 60 Å; (**c**) material extrusion width on the side of the workpiece at different indentation depths and positions; (**d**) material extrusion height on the side of the workpiece at different indentation depths and positions.

When performing nanoindentation experiments on the edge of a single-crystal silicon specimen using a diamond indenter, the amount of material extruded from the side of the specimen is modulated by the indentation depth. As illustrated in Figure 5(a1,b1), when the indentation depth is set to 1.5 nm, no obvious particle extrusion phenomenon is observed at the 80 Å indentation position; in contrast, at the 40 Å position, elliptical material extrusion occurs on the side of the specimen, accompanied by a slight collapse is at the edge in the -X direction. As illustrated in Figure 5(a2–a4,b2–b4), as the indentation depth increases, the amount of material extruded from the side of the specimen also shows an upward trend. When the indentation position is 60 Å and the depth exceeds 2.6 nm, a small collapse begins to appear at the edge of the indentation in the -X direction, and the width of the material extrusion is significantly higher than at 40 Å, while the height of extrusion is lower at 60 Å compared to 40 Å. At indentation depths greater than 2.6 nm, the collapse range and height at the -X direction edge of the indentation at 60 Å are also lower compared to 40 Å. Figure 5c,d present bar charts showing the variation in the width and height of material extrusion on the specimen side with different indentation depths and positions. When the indentation position is 120 Å, based on the results presented in Figure 5c,d, no material extrusion is observed at different indentation depths, indicating that edge effects are not present at this position. However, at indentation positions of 100 Å, 80 Å, 60 Å, and 40 Å, distinct differences in material extrusion on the specimen side are evident. As illustrated in Figure 5c,d, as the indentation position gradually approaches the edge of the specimen in the -X direction, the material extrusion phenomenon begins to appear at smaller indentation depths; moreover, both the width and height of the material extrusion increase with the increase in the indentation depth, which, in turn, leads to a more and more obvious edge effect.

### 3.2. Mechanical Properties

**Figure 6:**
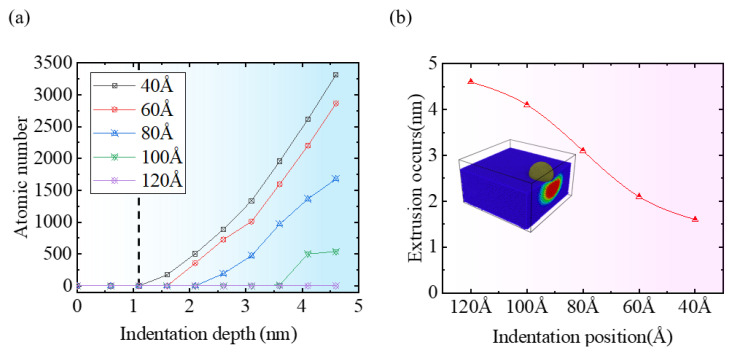
The changes in particle extrusion on the side of the workpiece at different indentation depths. (**a**) Curve of the number of particles extruded from the side of the workpiece as a function of indentation depth; (**b**) curve of the indentation depth at which particle extrusion occurs on the side of the workpiece for different indentation positions.

Figure 6a depicts the fluctuation in the number of material extrusion particles on the side of the specimen with indentation depth at different indentation positions; Figure 6b further elucidates the critical indentation depth at which material extrusion begins to appear on the side of the specimen at various indentation positions. According to Figure 6b, when the indentation position is set to 120 Å, no particle extrusion is observed on the specimen side, indicating that no edge effects are present at this indentation position. However, when the indentation position is 100 Å and the indentation depth is 3.6 nm, material extrusion begins to appear on the specimen side, indicating that slight edge effects start to emerge in the single-crystal silicon specimen at this point. Additionally, as the indentation position moves closer to the specimen edge, according to Figure 6a, the number of extrusion particles on the side of the specimen increases significantly, exhibiting a linear increasing trend for indentation positions less than 60 Å, indicating a clear edge effect. For indentation positions less than 100 Å, under the same indentation depth conditions, the number of extrusion particles on the side of the specimen significantly increases as the indentation position approaches the edge. When the indentation depth is below 1.1 nm, no particle extrusion is observed on the specimen side for different indentation positions. Furthermore, according to Figure 6b, as the indentation position gradually approaches the edge of the specimen, the depth at which material extrusion begins to occur on the side of the specimen also gradually decreases.

**Figure 7:**
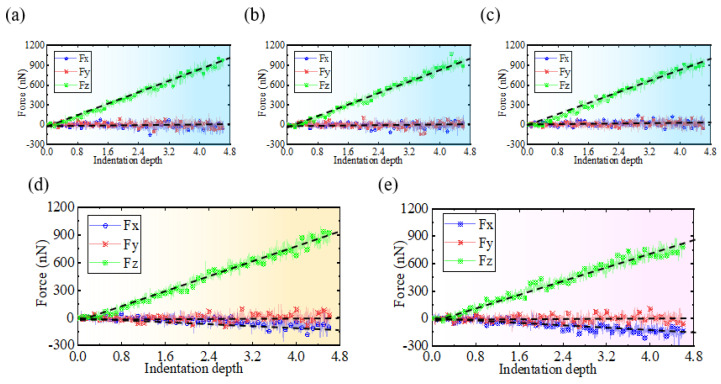
Changing trends in indentation force relative to indentation depth, observed across different indentation positions. The indentation positions for (**a**–**e**) are 120 Å, 100 Å, 80 Å, 60 Å, and 40 Å, respectively.

When performing nanoindentation experiments on the edge of single-crystal silicon using a diamond indenter, both the indentation depth and position exert a modulating effect on the indentation force. Figure 7a–e visually represent the trend of the indentation force changing with indentation depth at different indentation positions. As illustrated in Figure 7a–c, when the indentation position exceeds 80 Å, the vertical indentation force Fz exhibits a linear increasing trend with the increase in indentation depth, and when the indentation depth reaches 4.6 nm, its peak force value approaches 1050 nN, while the horizontal indentation forces *Fx* and *Fy* show no significant trend, fluctuating around zero and remaining stable. However, when the indentation position is less than or equal to 60 Å, as illustrated in Figure 7d,e, *Fz* starts to exhibit variations. The rate of the increase in Fz decreases as the indentation position moves closer to the specimen edge. Under the maximum indentation depth condition of 4.6 nm, the peak force values of *Fz* corresponding to the indentation positions of 80 Å and 60 Å are approximately 920 nN and 880 nN, respectively. Concurrently, the horizontal indentation force Fx also begins to exhibit an increasing trend along the negative X direction, with its peak force value being approximately −150 nN. At an indentation position of 40 Å, the fluctuation in *Fx* becomes more pronounced. For *Fy*, when the indentation position is less than 60 Å, the force remains around zero with a slight increase in fluctuation amplitude.

**Figure 8:**
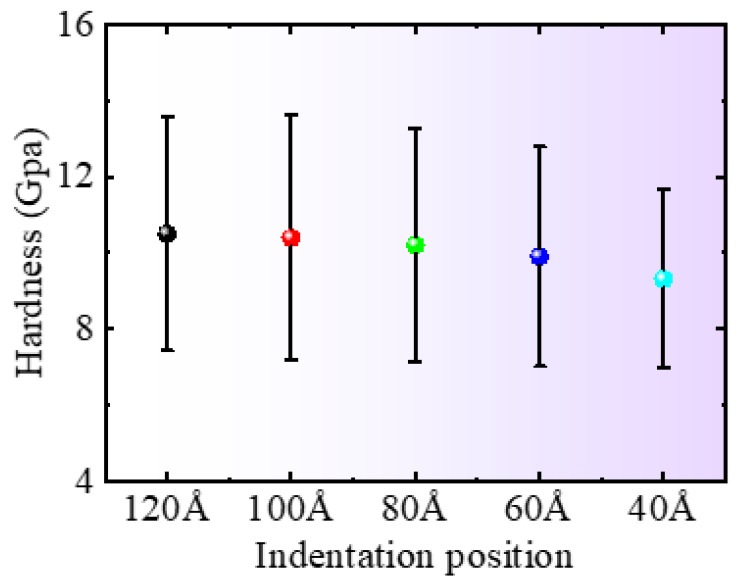
Mean and variance of hardness at different nanoindentation positions.

Figure 8 shows the changes in the average hardness and variance in single-crystal silicon at different indentation positions. As the indentation position gradually approaches the edge, the average hardness of the silicon decreases, indicating that the hardness of the material at the edge is low, which is also the reason for the decrease in the indentation force *Fz*. At the same time, regarding the variance in the hardness of single-crystal silicon, the magnitude of the change in material hardness changes with the depth of indentation. It can be seen that the hardness variance is highest at the indentation position of 100 Å and then gradually decreases to reach its minimum value at 40 Å, indicating that the hardness change in the material at 100 Å is relatively large, while the hardness change at the edge position is relatively small with the depth of indentation.

**Figure 9:**
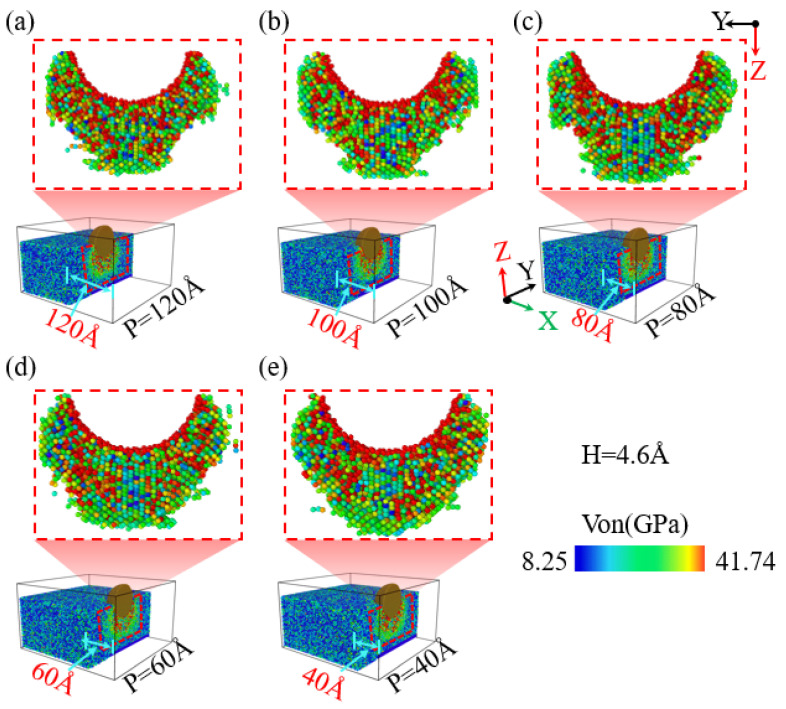
The von Mises stress distribution inside the YZ cross-section at different indentation positions with an indentation depth of 4.6 nm. (**a**–**e**) von Mises stress on YZ section at indentation positions of 120 Å, 100 Å, 80 Å, 60 Å, and 40 Å, respectively.

When performing nanoindentation experiments on the edge of single-crystal silicon using a diamond indenter, the indentation position significantly modulates the stress field distribution beneath the indenter. Figure 9a–e depict, in schematic diagram form, the influence of different indentation positions on the stress field distribution within the YZ plane of the specimen at an indentation depth of 4.6 nm. As illustrated in Figure 9a–c, when the indentation positions are 120 Å, 100 Å, and 80 Å, the distribution pattern of the stress beneath the indenter within the YZ plane is primarily concentrated directly beneath the indenter and on either side, and the distribution is relatively symmetrical. However, contrasting Figure 9d,e, it can be observed that as the indentation position approaches the edge of the specimen, at positions such as 60 Å and 40 Å, the distribution pattern of stress beneath the indenter within the YZ plane begins to shift towards the Y direction of the specimen. The high-stress regions also shift accordingly, with the distribution of high-stress areas becoming concentrated more in the Y direction beneath the indenter in the YZ plane. Additionally, referencing Figure 9a–e, it is evident that as the indentation position gradually approaches the edge of the specimen, both the range and depth of the stress distribution beneath the indenter within the YZ plane increase, while simultaneously, the depth of the high-stress region exhibits a progressively decreasing trend, this indicates that the phenomenon of edge stress concentration is consistent with the changes in the surface collapse morphology of the material. In addition, it can be observed that the stress does not propagate to the bottom of the material, and the fixed substrate is not affected by indentation. The simulation results are reliable.

**Figure 10:**
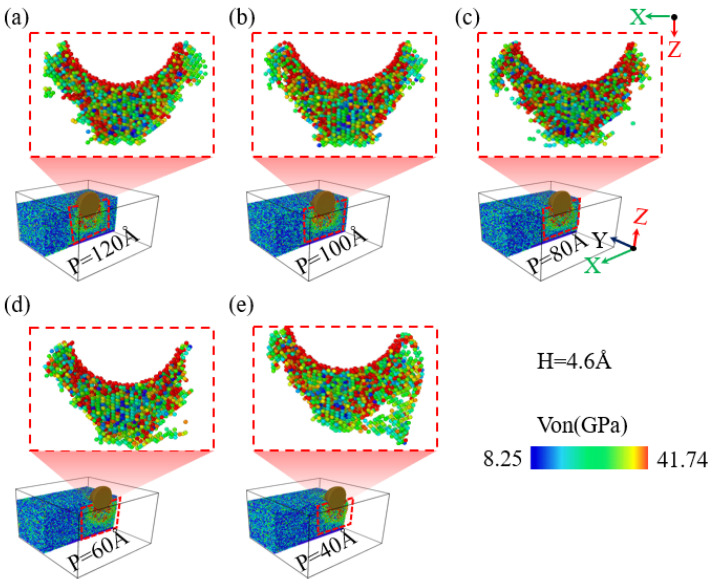
The von Mises stress distribution inside the XZ cross-section at different indentation positions with an indentation depth of 4.6 nm. (**a**–**e**) von Mises stress on XZ section at indentation positions of 120 Å, 100 Å, 80 Å, 60 Å, and 40 Å, respectively.

Figure 10 shows the stress distribution within the XZ plane of the specimen at an indentation depth of 4.6 nm for different indentation positions. According to Figure 10a, when the indentation position is set to 120 Å, the high-stress region beneath the indenter in the XZ plane is mainly gathered directly below the indenter. However, contrasting Figure 10b,c, it can be observed that as the indentation position approaches the edge of the specimen, at positions of 100 Å and 80 Å, the high-stress region distribution in the XZ plane beneath the indenter begins to shift towards the -X direction of the specimen. Despite this shift, the high-stress areas are still mainly concentrated beneath the indenter and on either side, maintaining a relatively symmetrical distribution. As illustrated in Figure 10d,e, with further movement of the indentation position towards the edge of the specimen, at positions of 60 Å and 40 Å, the high-stress region beneath the indenter in the XZ plane shows a significant increase in the shift towards the -X direction, this is consistent with the trend of material side extrusion changing with the indentation position. The distribution of the stress region becomes primarily concentrated in the -X direction beneath the indenter. Additionally, at an indentation position of 40 Å, the stress beneath the indenter in the XZ plane decreases, simultaneously, the proportional share of the stress region distribution in the -X direction exhibits a significantly increasing trend.

**Figure 11:**
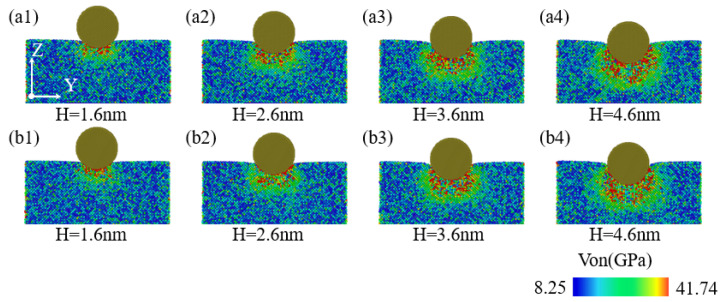
The von Mises stress analysis inside the YZ cross-section at different indentation depths. In the figure, (**a1**–**a4**) correspond to an indentation position of 40 Å, and (**b1**–**b4**) correspond to an indentation position of 60 Å.

When performing nanoindentation experiments on the edge of single-crystal silicon using a diamond indenter, the indentation depth exerts a crucial impact on the internal stress field distribution of the specimen. Figure 11(a1–a4,b1–b4) aim to illustrate the pattern of changes exhibited by the stress field distribution within the YZ plane of the specimen with indentation depth, specifically for indentation positions of 40 Å and 60 Å. As illustrated in Figure 11(a1,a2,b1,b2), when the indentation depth is less than or equal to 2.6 nm, the stress distribution and high-stress regions within the YZ plane are mainly concentrated directly beneath the indenter, with the stress distribution being symmetrical relative to the XZ plane. When the indentation depth is greater than or equal to 3.6 nm, as illustrated in Figure 11(a3,a4,b3,b4), the stress distribution within the YZ plane increases significantly with the indentation depth. The stress field distribution pattern and high-stress regions extend and distribute beneath the indenter and to both lateral sides, with the proportional share of stress regions being more pronounced in the -Y direction compared to the -X direction. Additionally, as illustrated in Figure 11(a4,b4), under an indentation depth of 4.6 nm, it is observed that the high-stress region distribution within the YZ plane shifts slightly more in the -Y direction for an indentation position of 40 Å compared to the distribution at 60 Å, with the distribution range being deeper.

**Figure 12:**
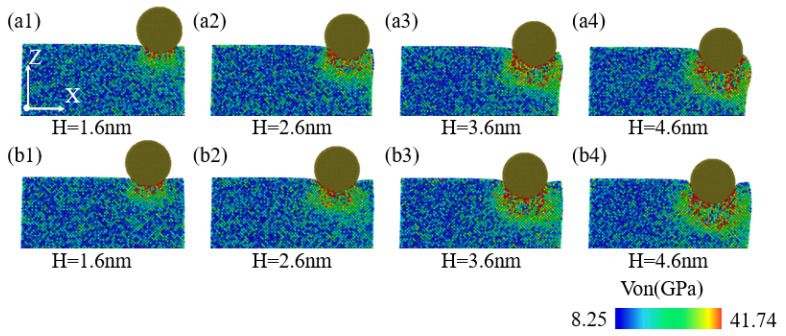
The von Mises stress analysis inside the XZ cross-section at different indentation depths. In the figure, (**a1**–**a4**) correspond to an indentation position of 40 Å, and (**b1**–**b4**) correspond to an indentation position of 60 Å.

Figure 12(a1–a4,b1–b4) aim to reveal the pattern of stress variation within the XZ plane of the specimen with indentation depth at indentation positions of 40 Å and 60 Å. Given an indentation depth of 1.6 nm, comparing Figure 12(a1,b1), it is evident that the stress field distribution pattern and high-stress regions within the XZ plane are mainly concentrated in the area directly beneath the indenter. As the indentation depth continuously increases, referring to Figure 1(a2–a4,b2–b4), for indentation positions of 40 Å and 60 Å, the stress level within the XZ plane also progressively rises, and the extent of the high-stress regions also expands accordingly. The high-stress regions are mainly concentrated directly beneath the indenter. Considering the influence of the specimen’s lateral face, the stress field distribution in the XZ plane exhibits a tendency to deviate towards the indenter’s -X direction. Additionally, as illustrated in Figure 12(a1–a4,b1–b4), when the indentation depth is less than or equal to 2.6 nm, the stress distribution within the XZ plane at indentation positions of 40 Å and 60 Å is essentially similar. However, when the indentation depth is no less than 3.6 nm, the stress distribution and extent of the high-stress regions within the XZ plane beneath the indenter for an indentation position of 60 Å are significantly greater compared to those for an indentation position of 40 Å.

### 3.3. Internal Phase Transition

**Figure 13:**
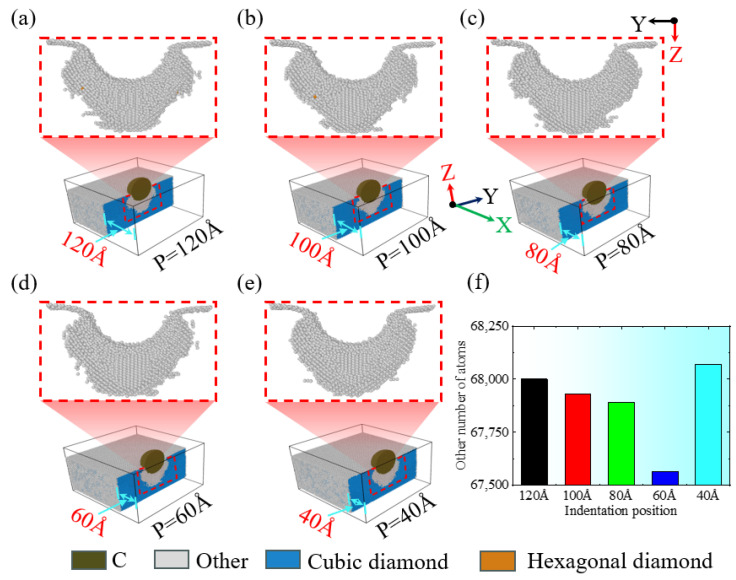
The defect distribution inside the YZ cross-section at different indentation positions with an indentation depth of 4.6 nm. (**a**–**e**) defect distribution in YZ section at indentation positions of 120 Å, 100 Å, 80 Å, 60 Å, and 40 Å, respectively; (**f**) Bar chart of other particle numbers.

When conducting nanoindentation experiments on the edge of single-crystal silicon using a diamond indenter, the indentation position influences the phase transformation phenomenon beneath the indenter. Figure 13a–e present, in the form of schematic diagrams, the characteristics of the phase transformation regions beneath the indenter in the YZ plane at an indentation depth of 4.6 nm for different indentation positions. The reason for the formation of this phase transition is that irreversible plastic deformation occurs at the material edge after nanoindentation, leading to the formation of an amorphous structure inside [50]. By comparing Figure 13a,b, it is evident that when the indentation positions are 120 Å and 100 Å, respectively, the distribution range and depth of the phase transformation region beneath the indenter in the YZ plane exhibit a high degree of similarity, primarily concentrated beneath and on either side of the indenter, with a small amount of hexagonal diamond structure observable at the edges of the phase transformation region. However, comparing Figure 13c–e, it is evident that as the indentation position moves closer to the specimen edge, at 80 Å, differences in the phase transformation region within the YZ plane become evident. Both the range and depth of the phase transformation region are expanded, with the distribution becoming more concentrated directly beneath the indenter and expanding further towards the edge accompanying the indentation position nearing the specimen boundary. Additionally, for different indentation positions, referencing Figure 13a–e, it is evident that the phase transformation regions beneath the indenter in the YZ plane exhibit a symmetrical distribution pattern with respect to the XZ plane. In addition, it can be observed that the phase transition does not propagate to the bottom of the material, and the fixed substrate is not affected by indentation. The simulation results are reliable. As shown in Figure 13f, as the indentation position gradually approaches the edge, the number of amorphous particles gradually decreases. However, when the indentation position is 40 Å, the number of amorphous particles suddenly increases. This indicates that during the process of the indentation head gradually approaching the edge, energy is mainly released by ordered phase transition, and the number of amorphous particles decreases. However, when it reaches the outermost position, the material boundary constraint collapses, and the amorphous phase dominates the deformation mechanism.

**Figure 14:**
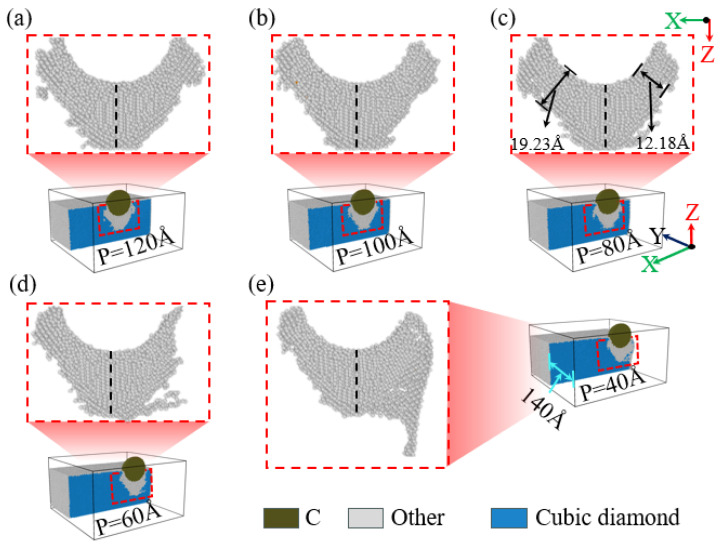
The defect distribution inside the XZ cross-section at different indentation positions with an indentation depth of 4.6 nm. (**a**–**e**) defect distribution in XZ section at indentation positions of 120 Å, 100 Å, 80 Å, 60 Å, and 40 Å, respectively.

Figure 14a–e show schematic diagrams of the phase transformation regions beneath the indenter in the XZ plane at an indentation depth of 4.6 nm for different indentation positions. Referring to Figure 14a,b, when the indentation positions are set to 120 Å and 100 Å, the distribution range and depth of the phase transformation region beneath the indenter in the XZ plane exhibit similarity, and both are mainly concentrated beneath the indenter, further manifesting a symmetrical distribution characteristic relative to the YZ plane. However, when the indentation position is 80 Å, as illustrated in Figure 14c, the distribution of the phase transformation region in the XZ plane starts to expand towards the X direction inside the specimen. Both the distribution range and depth of the phase transformation region are expanded, and slight asymmetry relative to the YZ plane also begins to manifest itself. In contrast, as illustrated in Figure 14d, when the indentation position moves closer to the specimen edge and is at 60 Å, the distribution of the phase transformation region in the XZ plane starts to extend towards the outer -X direction of the specimen and presents an irregular distribution, this trend indicates that the stacking behavior of side materials is influenced by phase transitions. Additionally, as illustrated in Figure 14e, when the indentation position is set to 40 Å, the main distribution direction of the phase transformation region beneath the indenter in the XZ plane is concentrated in the -X axial direction, and the distribution range also exhibits a significantly expanded state. However, the depth of the phase transformation region tends to become shallower, and its distribution pattern also exhibits significant asymmetry relative to the YZ plane.

**Figure 15:**
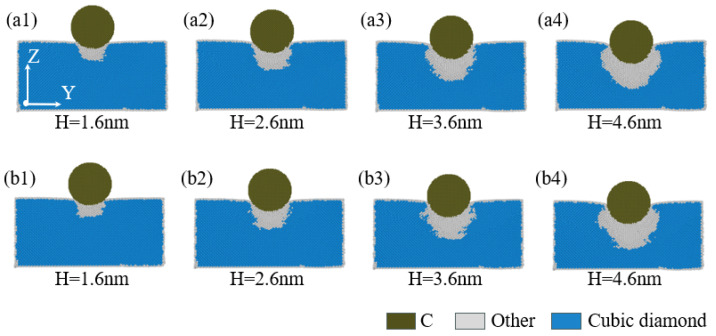
The defect distribution inside the YZ cross-section at different indentation depths. In the figure, (**a1**–**a4**) correspond to an indentation position of 40 Å, and (**b1**–**b4**) correspond to an indentation position of 60 Å.

When performing nanoindentation experiments on the edge of single-crystal silicon using a diamond indenter, the indentation depth exerts an action effect on the phase transformation phenomenon beneath the indenter contact area. Referencing Figure 15(a1,b1), it is evident that at an indentation depth of 1.6 nm, and under indentation positions set at 40 Å and 60 Å, respectively, the distribution pattern of the phase transformation region beneath the indenter in the YZ plane is similar, with the phase transformation area being relatively small and primarily concentrated directly beneath the indenter. As the indentation depth continues to increase, as depicted in Figure 15(a2,b2), when the depth reaches 2.6 nm, the distribution range of the phase transformation region beneath the indenter in the YZ plane also exhibits a significant expansion with further indentation deepening. At the 40 Å indentation position, a slight displacement of the phase transformation region along the -Y direction exists. However, with further progressive increases in indentation depth, comparing Figure 15(a3,a4,b3,b4), it is evident that when the depth is no less than 3.6 nm, the distribution range of the phase transformation region in the YZ plane then exhibits a clear expansion. The phase transformation region then begins to gather directly beneath the indenter and on both sides, and the distribution of the phase transformation region also exhibits a pattern approximately symmetrical with the XZ plane.

**Figure 16:**
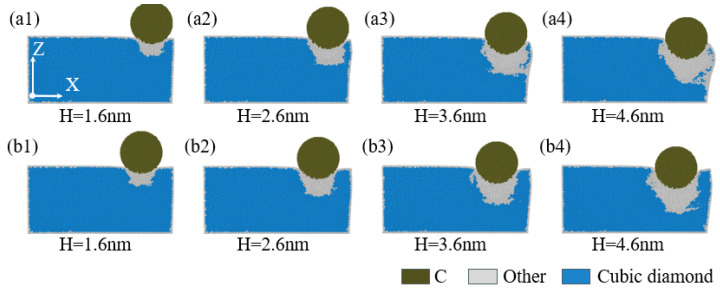
The defect distribution inside the XZ cross-section at different indentation depths. In the figure, (**a1**–**a4**) correspond to an indentation position of 40 Å, and (**b1**–**b4**) correspond to an indentation position of 60 Å.

Figure 16(a1–a4,b1–b4) show schematic diagrams of the phase transformation regions beneath the indenter in the XZ plane at indentation positions of 40 Å and 60 Å as a function of indentation depth. As illustrated in Figure 16(a1,b1), at an indentation depth of 1.6 nm, the phase transformation regions beneath the indenter in the XZ plane at both 40 Å and 60 Å are similar, with a relatively small distribution range and the phase transformation region concentrated directly beneath the indenter, symmetrically distributed relative to the YZ plane. However, when the indentation depth reaches 2.6 nm, comparing Figure 16(a2,b2), it is evident that both the distribution range and depth of the phase transformation region beneath the indenter in the XZ plane are expanded, and their distribution pattern also begins to shift towards the -X direction of the specimen. This shift causes slight asymmetry relative to the YZ plane. Accompanying the subsequent increase in indentation depth, referring to Figure 16(a3,a4,b3,b4), when the depth is no less than 3.6 nm, for both 40 Å and 60 Å indentation positions, the distribution range and depth of the phase transformation region beneath the indenter in the XZ plane both achieve significant enhancement. At larger indentation depths, the shift of the distribution of the phase transformation region in the XZ plane towards the -X direction becomes increasingly pronounced, and this region mainly converges beneath the indenter and tends towards the boundary edge of the specimen in the -X direction, thereby exhibiting an asymmetry characteristic relative to the YZ plane.

## 4. Conclusions

This study employs molecular dynamics simulation technology, aiming to explore the edge effect exhibited in the nanoindentation process at varying distances approaching the edge of a single-crystal silicon workpiece. The results indicate that as the indentation position approaches the edge of the workpiece, the collapse range at the indentation becomes wider, and more atoms are extruded from the side of the workpiece. Simultaneously, the shape of the lateral extrusion also exhibits an elliptical form, a characteristic closely related to the crystal structure of the material. In terms of the indentation force, the indentation position has the greatest impact on Fz. Accompanying the indentation position converging towards the workpiece boundary, Fz is smaller at 80 Å and 60 Å, attributable to the hardness of the material edge region being lower compared to the central region. The von Mises stress distribution and phase transition area inside the workpiece are mainly concentrated below the indenter. Accompanying the indentation position shifting towards the workpiece boundary, phase transition also occurs on the side of the indenter near the edge of the workpiece, which means that an edge effect is generated. At present, there is relatively little experimental research on the edge effect of single-crystal silicon nanoindentation. This study employs molecular dynamics simulation methodology to thoroughly investigate the edge effect of single-crystal silicon nanoindentation, with the aim of offering a reference for related experiments, aiding in the effective implementation of nanoindentation experiments, and deepening the understanding of the characteristics of single-crystal silicon material.

## Figures and Tables

**Table 1:** Simulation parameters for single-crystal Si nanoindentation.

Nanoindentation Parameters	Value
Workpiece material	Single-crystal Si, lattice constant 5.43 Å
Workpiece dimensions (nm^3^)	28 × 28 × 13
Tool material	Diamond with a 3.57 Å lattice constant
Tool radius (nm)	4
Boundary condition	SPS
Potential function	Tersoff
Nanoindentation speed (m/s)	50
Nanoindentation depth (nm)	4.6
Nanoindentation position (Å)	120, 100, 80, 60, 40
Simulation ensemble	LAMMPS
Atomic quantity	561,304
Workpiece temperature (K)	293
Timestep (fs)	1

## Data Availability

The original contributions presented in the study are included in the article, further inquiries can be directed to the corresponding author.

## References

[1] Sun J., Cheng L., Han J., Ma A., Fang L. (2017). Nanoindentation Induced Deformation and Pop-in Events in a Silicon Crystal: Molecular Dynamics Simulation and Experiment. Sci. Rep..

[2] Du X., Zhao H., Zhang L., Yang Y., Xu H., Fu H., Li L. (2015). Molecular dynamics investigations of mechanical behaviours in monocrystalline silicon due to nanoindentation at cryogenic temperatures and room temperature. Sci. Rep..

[3] Chen J., Shi J., Zhang M., Peng W., Fang L., Sun K., Han J. (2018). Effect of indentation speed on deformation behaviors of surface modified silicon: A molecular dynamics study. Comput. Mater. Sci..

[4] Dastgeer G., Nisar S., Zulfiqar M.W., Eom J., Imran M., Akbar K. (2024). A Review on Recent Progress and Challenges in High-Efficiency Perovskite Solar Cells. Nano Energy.

[5] Dai H., Zhang F., Zhou Y., Chen J. (2019). Numerical study of three-body diamond abrasive nanoindentation of single-crystal Si by molecular dynamics simulation. Appl. Phys. A Mater. Sci. Process.

[6] Goel S., Faisal N.H., Luo X., Yan J., Agrawal A. (2014). Nanoindentation of polysilicon and single crystal silicon: Molecular dynamics simulation and experimental validation. J. Phys. D Appl. Phys..

[7] Sun J., Xu B., Zhuo X., Han J., Yang Z., Jiang J., Ma A., Wu G., Chu P.K. (2020). Investigation of Indenter-Size-Dependent Nanoplasticity of Silicon by Molecular Dynamics Simulation. ACS Appl. Electron. Mater..

[8] Lin Y.-H., Jian S.-R., Lai Y.-S., Yang P.-F. (2008). Molecular dynamics simulation of nanoindentation-induced mechanical deformation and phase transformation in monocrystalline silicon. Nanoscale Res. Lett..

[9] Dastgeer G., Nisar S., Rasheed A., Akbar K., Chavan V.D., Kim D.-K., Wabaidur S.M., Zulfiqar M.W., Eom J. (2023). Atomically Engineered, High-Speed Non-Volatile Flash Memory Device Exhibiting Multibit Data Storage Operations. Nano Energy.

[10] Yaghoobi M., Voyiadjis G.Z. (2014). Effect of boundary conditions on the MD simulation of nanoindentation. Comput. Mater. Sci..

[11] Yan C., Bor B., Plunkett A., Domènech B., Maier-Kiener V., Giuntini D. (2023). Nanoindentation Creep of Supercrystalline Nanocomposites. Mater. Des..

[12] Cho H., Lee J., Hwang H., Hwang W., Kim J.-G., Kim S. (2022). Mechanical Properties of Graphene Oxide–Silk Fibroin Bionanofilms via Nanoindentation Experiments and Finite Element Analysis. Friction.

[13] Schuh C.A. (2006). Nanoindentation studies of materials. Mater. Today.

[14] Zhang Z., Zhang Z., Zhao D., Niu Y., Bai D., Wang Y., Song M., Zhao J., Wang S., Zhu B. (2024). Effect of temperature on the nanoindentation behavior of monocrystalline silicon by molecular dynamics simulations. Mater. Today Commun..

[15] Gannepalli A., Mallapragada S.K. (2001). Molecular dynamics studies of plastic deformation during silicon nanoindentation. Nanotechnology.

[16] Zarudi I., Zou J., Zhang L.C. (2003). Microstructures of phases in indented silicon: A high resolution characterization. Appl. Phys. Lett..

[17] Bradby J.E., Williams J.S., Wong-Leung J., Swain M.V., Munroe P. (2000). Transmission electron microscopy observation of deformation microstructure under spherical indentation in silicon. Appl. Phys. Lett..

[18] Zhao H., Shi C., Zhang P., Zhang L., Huang H., Yan J. (2012). Research on the effects of machining-induced subsurface damages on mono-crystalline silicon via molecular dynamics simulation. Appl. Surf. Sci..

[19] Lin Y.-H., Chen T.-C. (2008). A molecular dynamics study of phase transformations in mono-crystalline Si under nanoindentation. Appl. Phys. A Mater. Sci. Process.

[20] Zhao Z., Zhou S., Li X., Zhu B., Guan S., Wang S., Zhao H. (2024). Investigation of cracking in monocrystalline silicon induced by high- temperature indentation. Eng. Fail. Anal..

[21] Heidari M., Karimzadeh A., Ayatollahi M., Yahya M. (2021). Effects of Particle Distribution and Calculation Method on Results of Nano-Indentation Technique in Heterogeneous Nanocomposites-Experimental and Numerical Approaches. Int. J. Solids Struct..

[22] Goel S., Kovalchenko A., Stukowski A., Cross G. (2016). Influence of microstructure on the cutting behaviour of silicon. Acta Mater..

[23] Sun J., Fang L., Han J., Han Y., Chen H., Sun K. (2014). Phase transformations of mono-crystal silicon induced by two-body and three-body abrasion in nanoscale. Comput. Mater. Sci..

[24] Sun J., Fang L., Han J., Han Y., Chen H., Sun K. (2013). Abrasive wear of nanoscale single crystal silicon. Wear.

[25] Mylvaganam K., Zhang L. (2011). Nanotwinning in monocrystalline silicon upon nanoscratching. Scr. Mater..

[26] Zimmerman J.A., Kelchner C.L., Klein P.A., Hamilton J.C., Foiles S.M. (2001). Surface step effects on nanoindentation. Phys. Rev. Lett..

[27] Kelchner C.L., Plimpton S.J., Hamilton J.C. (1998). Dislocation nucleation and defect structure during surface indentation. Phys. Rev. B.

[28] Li J., Van Vliet K.J., Zhu T., Yip S., Suresh S. (2002). Atomistic mechanisms governing elastic limit and incipient p1asticity in crystals. Nature.

[29] Lee Y., Park J.Y., Kim S.Y., Jun S., Im S. (2005). Atomistic simulations of incipient plasticity under Al(111) nanoindentation. Mech. Mater..

[30] Jang H., Farkas D. (2007). Interaction of lattice dislocations with a grain boundary during nanoindentation simulation. Mater. Lett..

[31] Nair A.K., Parker E., Gaudreau P., Farkas D., Kriz R.D. (2008). Size effects in indentation response of thin films at the nanoscale: A molecular dynamics study. Int. J. Plast..

[32] Lin Y.-H., Chen T.-C., Yang P.-F., Jian S.-R., Lai Y.-S. (2007). Atomic-level simulations of nanoindentation-induced phase transformation in mono-crystalline silicon. Appl. Surf. Sci..

[33] Chung Y.J., Lee G.H., Beom H.G. (2022). Atomistic Insights into the Phase Transformation of Single-Crystal Silicon during Nanoindentation. Nanomaterials.

[34] Jiao S., Huang Q., Tu W., Chen J., Sun Z. (2019). Investigation on the phase transformation of monocrystalline silicon during nanoindentation at cryogenic temperature by molecular dynamics simulation. Phys. B Condens. Matter.

[35] Ge G., Rovaris F., Lanzoni D., Barbisan L., Tang X., Miglio L., Marzegalli A., Scalise E., Montalenti F. (2024). Silicon phase transitions in nanoindentation: Advanced molecular dynamics simulations with machine learning phase recognition. Acta Mater..

[36] Li Q. (2023). Edge effect and indentation depth-dependent contact behavior in contact of an elastic quarter-space. Int. J. Solids Struct..

[37] Jakes J.E., Stone D.S. (2011). The edge effect in nanoindentation. Philos. Mag..

[38] Liu H., Zhao P., Zhu W., Pan J., Wang Z., Gao X., Wang S., Tan J. (2024). Investigation of edge effect on wurtzite gallium nitride in nanoindentation using molecular dynamics simulation. Mater. Today Commun..

[39] Li Z., Kang S., Wu D., Ren M., Zhang X., Lei J., Zhu L., Li D. (2024). The effect of temperature on monocrystalline Si nanoindentation side effects: A molecular dynamics study. Vacuum.

[40] Wang Y., Tang S., Guo J. (2020). Molecular dynamics study on deformation behaviour of monocrystalline GaN during nano abrasive machining. Appl. Surf. Sci..

[41] Nosé S. (1984). A unified formulation of the constant temperature molecular dynamics methods. J. Chem. Phys..

[42] Khan H.M., Kim S.-G. (2011). On the wear mechanism of thin nickel film during AFM-based scratching process using molecular dynamics. J. Mech. Sci. Technol..

[43] Tersoff J. (1988). New empirical approach for the structure and energy of covalent systems. Phys. Rev. B.

[44] Zhang J., Zhang J., Wang Z., Hartmaier A., Yan Y., Sun T. (2017). Interaction between phase transformations and dislocations at incipient plasticity of monocrystalline silicon under nanoindentation. Comput. Mater. Sci..

[45] Herbert E., Pharr G., Oliver W., Lucas B., Hay J. (2001). On the measurement of stress–strain curves by spherical indentation. Thin Solid Films.

[46] Chen C., Li H., Xiang H., Peng X. (2018). Molecular Dynamics Simulation on B3-GaN Thin Films under Nanoindentation. Nanomaterials.

[47] Plimpton S. (1995). Fast Parallel Algorithms for Short-Range Molecular Dynamics. J. Comput. Phys..

[48] Stukowski A. (2010). Visualization and analysis of atomistic simulation data with OVITO—The Open Visualization Tool. Model. Simul. Mater. Sci. Eng..

[49] Li R., Li Z., Kang S., Long C., Liu H., Zhao P., Li D. (2025). Molecular Dynamics Study on the Edge Effect of Single Crystal Ni with Two Indenters Nanoindentation. Vacuum.

[50] Trachet A., Subhash G. (2016). Microscopic and Spectroscopic Investigation of Phase Evolution Within Static and Dynamic Indentations in Single-Crystal Silicon. Mater. Sci. Eng. A.

